# Snapshot Sampling May Not Be Enough to Obtain Robust Estimates for Riverine Microplastic Loads

**DOI:** 10.1021/acsestwater.4c00176

**Published:** 2024-04-12

**Authors:** Anna Kukkola, Uwe Schneidewind, Lee Haverson, Liam Kelleher, Jennifer D. Drummond, Gregory Sambrook Smith, Iseult Lynch, Stefan Krause

**Affiliations:** † School of Geography, Earth and Environmental Sciences, University of Birmingham, Edgbaston, Birmingham B15 2TT, United Kingdom; ‡ LEHNA - Laboratoire d’ecologie des hydrosystemes naturels et anthropises, University of Lyon, Darwin C & Forel, 3-6 Rue Raphaël Dubois, 69622 Villeurbanne, France; § Institute of Global Innovation, University of Birmingham, Birmingham B15 2SA, United Kingdom

**Keywords:** sampling frequency, campaign, WWTP, temporal, distribution

## Abstract

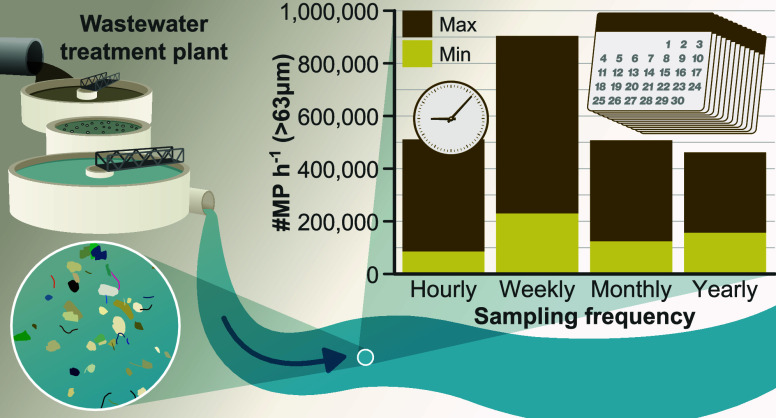

Wastewater treatment plants (WWTPs) have been described as key contributors of microplastics (MPs) to aquatic systems, yet temporal fluctuations in MP concentrations and loads downstream are underexplored. This study investigated how different sampling frequencies (hourly, weekly, and monthly) affect MP estimates in a stream linked to a single WWTP. Utilizing fluorescence microscopy and Raman spectroscopy, considerable hourly variations in MP concentrations were discovered, while the polymer composition remained consistent. This temporal variability in MP loads was influenced by MP concentration, discharge rates, or a mix of both. These results show a high uncertainty, as relying on sparse snapshot samples combined with annual discharge data led to significant uncertainties in MP load estimates (over- and/or underestimation of emissions by 3.8 billion MPs annually at this site). Our findings stress the necessity of higher-frequency sampling for better comprehending the hydrodynamic factors influencing MP transport. This improved understanding enables a more accurate quantification of MP dynamics, crucial for downstream impact assessments. Therefore, preliminary reconnaissance campaigns are essential for designing extended, representative site-monitoring programs and ensuring more precise trend predictions on a larger scale.

## Abstract 2

This study shows that the time and frequency of microplastic sampling downstream of a wastewater treatment plant considerably influence the concentration and load estimations, suggesting that high-frequency sampling should be applied for monitoring.

## 1 Introduction

Microplastics (MPs) are small plastic particles (<5 mm)^1^ that are found in all environmental compartments,^2−5^ including riverine environments.^6−9^ Evidence suggests that MPs can adsorb environmental pollutants with the potential for biomagnification.^10−12^ In addition, MPs can host distinct microbial communities in comparison to their immediate surroundings and are favorable substrates for known human pathogens, such as *Arcobacter*,^13,14^ making the study of the riverine transport and fate of MPs imperative. As a consequence, a growing body of research has been exploring the spatial distribution of MPs in various riverine environments and identifying drivers for MP transport such as discharge, sinuosity, and hydrometeorological events.^15−22^ The need to quantify the MP spatial variability across rivers and catchments has also been gaining recognition,^7,15,23,24^ and robust MP load estimates have been identified as being crucial for predictive modeling aiming to improve our understanding of the MP downstream fate and transport as well as MP distribution within wider river networks.^25,26^

Riverine MPs typically originate from a variety of point and diffuse sources.^27−29^ Wastewater treatment plants or sewage treatment works (hereafter WWTPs) can feature one major point source of input of MPs into river systems. Despite observed removal efficiencies of >90% (>300 μm),^30−33^ there is concern that the smaller MP fraction (<150 μm) escapes into the aquatic environment, even from WWTPs with a tertiary treatment stage.^34,35^ A recent review focusing on 38 different WWTPs across 11 countries estimated that the average daily MP emission from a typical WWTP amounts to 5.00 × 10^5^ to 1.39 × 10^10^ MPs, depending on the treatment plant design and population serving size.^31^ However, such load estimates are frequently based on extrapolations from snapshot sampling campaigns (i.e., mostly one sample or a few samples at most) that provide sparse and nontargeted data on MP concentrations, which are then upscaled in time, e.g., by combining them with average daily or annual discharge information.^31,36^ To date, few studies have examined the temporal variability in WWTP effluent,^37−42^ and such studies have typically restricted their analyses to a limited number of samples, for example, representing wet and dry seasons or 24 h composites, and overlooking the impact of daily release patterns.^37,38^ This lack of combined long-term and high-frequency data limits our understanding of potential temporal shifts in MP fluxes (loads). We hypothesize that incorporating data from various sampling intervals will enhance our understanding of riverine MP load variations that can be attributed to sporadic and irregular WWTP discharge patterns. Such integration will also provide more robust estimates of local downstream MP export as well as global plastic budgets and help us improve existing, or develop new, transport models for regional and global scales.^43,44^

Here, time-varying MP concentration data and quantification of MP loads for the Eastcote Brook, U.K., downstream from a WWTP are discussed. The WWTP effluent represents the sole water source and is the only significant contributor of MPs at the sampling location as there is no flow upstream of the WWTP. The data discussed here allow us (i) to quantify the short- to long-term variability in MP concentration and load in a stream with a known MP point source, (ii) to evaluate the impact of hydrological controls (discharge) on downstream MP transport, and (iii) to study the influence of different sampling intervals (monthly, weekly, and hourly) on MP load estimates from a single point source. The results improve our understanding of MP transport in dynamic river systems and can aid in the planning of future MP surface water sampling campaigns.

The aim of this study is also to contribute to developing and adopting more standardized and thus comparable MP sampling techniques and schemes for the release of MPs from WWTPs. Existing guidelines like the ISO 5667 series offer best practices for sampling aquatic contaminants, recognizing that frequent sampling improves estimates of dissolved loads.^45−48^ However, specific protocols for MP lag behind despite ongoing efforts to establish such standards.^49^ The newly introduced ISO 24187:2023 (Principles for the analysis of microplastics present in the environment), while a step forward, largely draws from ISO 5667 and cautions against direct method comparison,^50^ highlighting the unique behavior of MPs compared to soluble pollutants. The study presented here contributes crucial data to refine and support these emerging guidelines for MP.

## 2 Materials and Methods

### 2.1 Study Site

**Figure 1:**
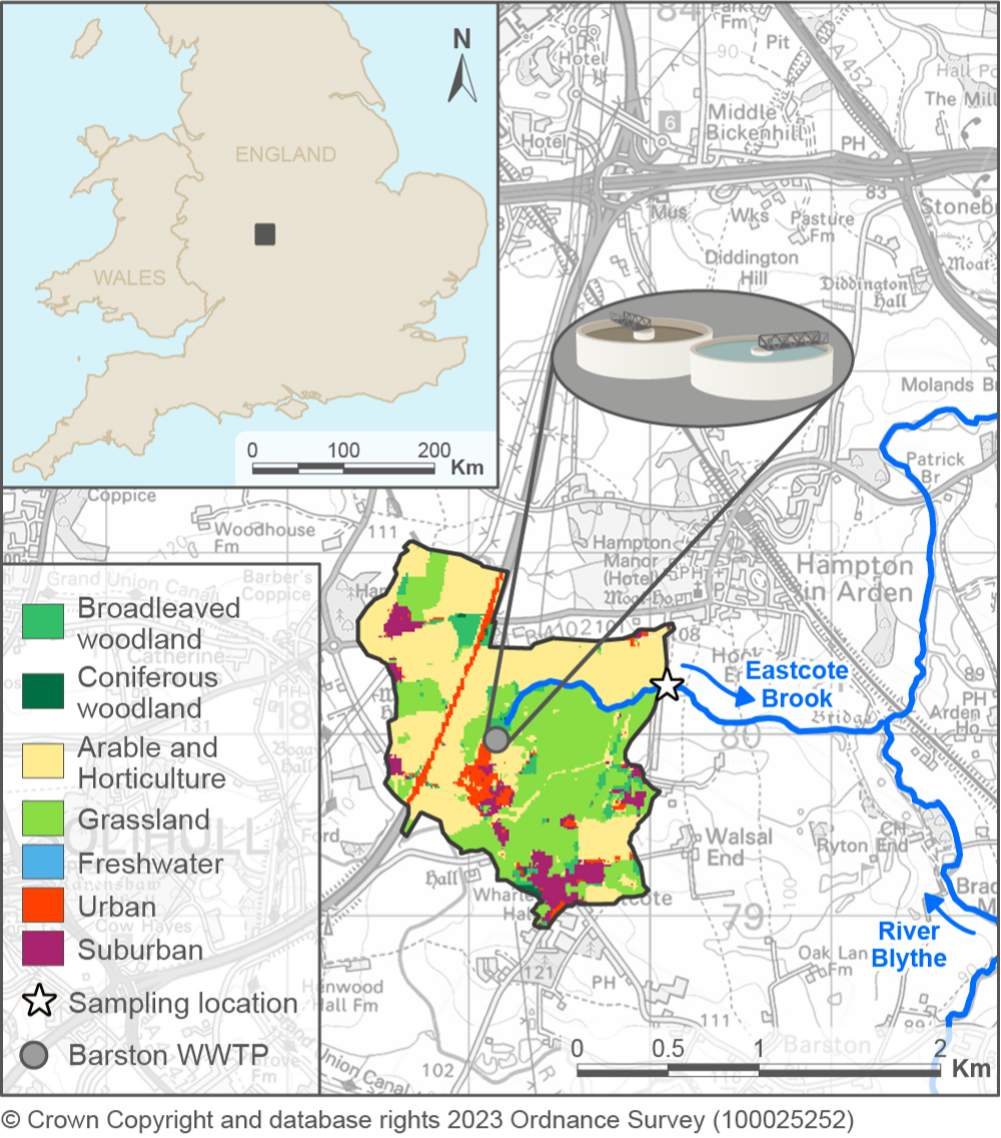
Map showing the sampling location (white star) near Birmingham, U.K., and its catchment (marked with a black line) with the major land use types. The Barston Wastewater Treatment Plant (WWTP) is indicated by the gray dot, and the blue arrows indicate the streamflow direction.

The sampling site (latitude, 52.4197; longitude, -1.7051) is located in a small partly channelized tributary to the River Blythe called Eastcote Brook (Figure 1), with a drainage area of 2.06 km^2^. Dominant land use in the catchment is agri- and horticulture (41.9%), followed by grassland (32.8%) and suburban and urban (12.9%), which comprises some light residential areas and the Barston Wastewater Treatment Plant (WWTP). The River Blythe later flows into the River Tame and then the River Trent, ultimately draining into the North Sea. According to historical maps, Eastcote Brook originates at Barston WWTP, a previous marshland area, and no streamflow exists upstream of the WWTP (confirmed by the WWTP operator and suggested by the hydrograph in Supporting Information S1, Hydrograph for discharge). Barston WWTP is equipped with a proprietary tertiary treatment system and sand filtration operated by Severn Trent Plc. and serves a population of roughly 62 500 across several hydrological catchments. Influent water arrives from urbanized regions of South Birmingham and Coleshill. The WWTP includes a storm treatment stream (395 L s^–1^ full flow to treatment), inlet screens, grit removal followed by a proprietary treatment system NEREDA, ferric dosing, and a Mecana cloth filter. The sampling site was located ∼1 km downstream of the WWTP (Figure 1), as access directly to the WWTP was not permitted, and areas further upstream of the sampling site proved to be inaccessible. At the sampling site, the stream was <3.6 m wide and <1.0 m deep. The riverbanks were covered in thick vegetation with trees also covering part of the water surface in the channel. Minimum, maximum, and mean WWTP effluent discharge were provided as 15 min interval data (liters per second) for the period between June 14 and 20, 2021, by Severn Trent Plc. (Supporting Information S1, Hydrograph for discharge). Additional WWTP effluent data to cover the entire year of sampling had been requested but not made available.

### 2.2 Field Sampling

A surface water sampling campaign was conducted over the course of one year (between April 1, 2021, and March 31, 2022) and consisted of 12 monthly (collected in the last week of each month in general) and eight weekly sampling dates (June 2, 8, 15, and 22 and July 2, 9, 15, and 26, 2021). Additionally, a high-frequency sampling campaign over 12 h on 4 days was conducted, with 1 h interval sampling, comprising two weekdays (Tuesday and Wednesday, June 15 and 16, 2021, respectively) and two weekend days (Saturday and Sunday, June 19 and and 20, 2021, respectively) during a dry spell. All samples, with the exception of hourly samples, were consistently collected between 9 a.m. and 11 a.m. in an attempt to reduce any potential time-dependent fluctuation related to the operations of the WWTP. On June 15, 16, 19, and 20, 2021, the surface water samples were collected every hour between 9 a.m. and 8 p.m., resulting in 12 samples for each of the four sampling days. Monthly sampling was selected to reflect a common practice, whereby snapshot sampling is used to describe and upscale what is happening in a system at a given time to encompass the whole annual cycle. Monthly samples were collected toward the end of each month (Supporting Information S2, Metadata for sampling events).

During each sampling, 50 L of surface water was collected from the center of the river from the upper 20 cm of the water column using a 2 L polypropylene (PP) jug. The water was filtered *in situ* through a 63 μm sieve (10 cm diameter) containing a nylon mesh, from which the contents were backwashed into 20 mL borosilicate glass vials using deionized (DI) water. All samples were collected in triplicate. The volume of water collected was based on the results of a pilot study and was chosen to provide a balance between capturing the time-variable MP concentration pattern and preventing clogging of the mesh with organic matter while achieving time sensitive sampling. All samples collected in this study (*n* = 204) were stored in a cooler and transported immediately after collection to the University of Birmingham where they were stored in a cold dark room at 6 °C before being processed.

Additional parameters measured *in situ* included the streamflow velocity and electrical conductivity (EC). The flow velocity (*n* = 36) was obtained using a Sensa-RC2 (Aqua Data Services Ltd.) electromagnetic velocity meter, from a predetermined and marked cross section of the stream. For each 30 cm subsection (beginning at 0, marking the edge of the bank), depth measurements were taken at the middle of the sections and coupled with the average velocity measurement (averaged over 15 s) that was obtained by moving the velocity meter steadily up and down the water column between 20% and 80% of the stream depth. The total cross-sectional stream discharge, *Q*_tot_ (cubic meters per second), was then calculated as the sum of the discharge measured in each subsection *Q*_*x*_, as follows:
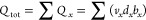
where *x* indicates the subsection, *v* is the flow velocity (meters per second), *b* is the subsection width (meters), and *d* is the subsection depth (meters). The electrical conductivity (EC) of the streamwater was monitored ∼50 m downstream of the sampling point every 15 min using a Solinst level logger. EC data (Supporting Information S2, Metadata for sampling events), however, are not available for the six samples from October 28, 2021, to March 23, 2022, due to loss of the logger.

### 2.3 Laboratory Analysis

#### 2.3.1 Sample Preparation, Digestion, and Staining

MPs collected as described in section 2.2 were extracted from the surface water samples following the protocol described by Kukkola et al.^7^ For details, see Supporting Information S3 (Sample preparation, digestion, and staining).

#### 2.3.2 Microscopy and Polymer Identification

Each filter was observed under fluorescence mode with a Macro zoom microscope (Olympus MVX-ZB10) with the settings discussed in refs (7) and (51). For details of configurations and detailed methods, see Supporting Information S4 (Microscopy and spectroscopy) and (52). For the detailed quality assurance/quality control (QA/QC) measures and recovery rates, see Supporting Information S5 (QA/QC procedures).

### 2.4 Data Analysis

The normality of all of the data was assessed using Shapiro–Wilk’s test, and nonparametric tests were applied where data were not normally distributed. To evaluate whether MP size (measured as the longest length) or MP concentration correlated with the average streamflow velocity at the sampling site (taken as the average of the three 30 cm midsections, which covered the stream segment where surface water samples were collected), stream total discharge, or EC, the nonparametric Spearman rank coefficient (*R*_s_) was used to assess the direction and strength of any correlation.

To compare MP concentrations between sampling dates with the same interval (monthly, weekly, and hourly), Kruskal–Wallis tests were applied, followed by the Dunn test with the Benjamini–Hochberg procedure to reduce false discovery rates. To assess whether any of the hourly triplicates were significantly different from the total daily mean, an unpaired two-sample Wilcoxon test was carried out. A Student’s *t* test was applied to assess the statistical significance of differences between MP concentrations and stream discharge between weekdays and weekend days. Statistical analyses were carried out in RStudio (RStudio, Inc., R Core Team, 2022). The significance threshold (α) was set to 0.05. All values for MP concentrations are reported with the mean and standard deviation (SD). The MP loads were calculated as
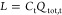
where *L* is the load (MPs per second), *C* is the MP concentration in the surface water (MPs per liter), *Q* is the discharge (cubic meters per second), and *t* is the time/date of the measurement. Although this calculation assumes a homogeneous MP distribution for the whole river cross section, it is possible that different MP concentrations for different polymer types would have been found in different subsections of the river with different flow properties and/or depths.^54^ As such, the loading rates shown in this study most closely represent surface loading rates (collected in the high-flow section of the river within the top 20 cm of the surface water column, with the river being <1 m deep), while depth-integrated concentrations/loads that include data from the wash zone or from near the stream bed might be different.

## 3 Results

### 3.1 Microplastic Particle Characteristics

MPs were identified in all samples collected during the observation period. Overall, clear/colorless was the most frequently assigned color-type category recorded (46.3% of total) and stained/pink was the second most dominant category (21.7%), followed by black (9.7% that were exclusively fibers) and white (5.0%). The color composition of different MPs did not correlate with the sampling frequency (Supporting Information S6, Microplastic colours per sampling frequency). Fragments represented the dominant morphology type of observed MPs (73.2%) with 26.5% being fibers and only 0.3% spheres, though consideration needs to be given to the fact that recovery for fibers was lower, and thus some fibers may have been lost during sampling and sample processing. A similar composition of MP morphologies was observed for the monthly and hourly samples; however, the weekly samples revealed an increase in the relative abundance of fibers from 33.3% at the end of June to 57.1% at the end of July (Supporting Information S7, Microplastic morphology distribution).

**Figure 2:**
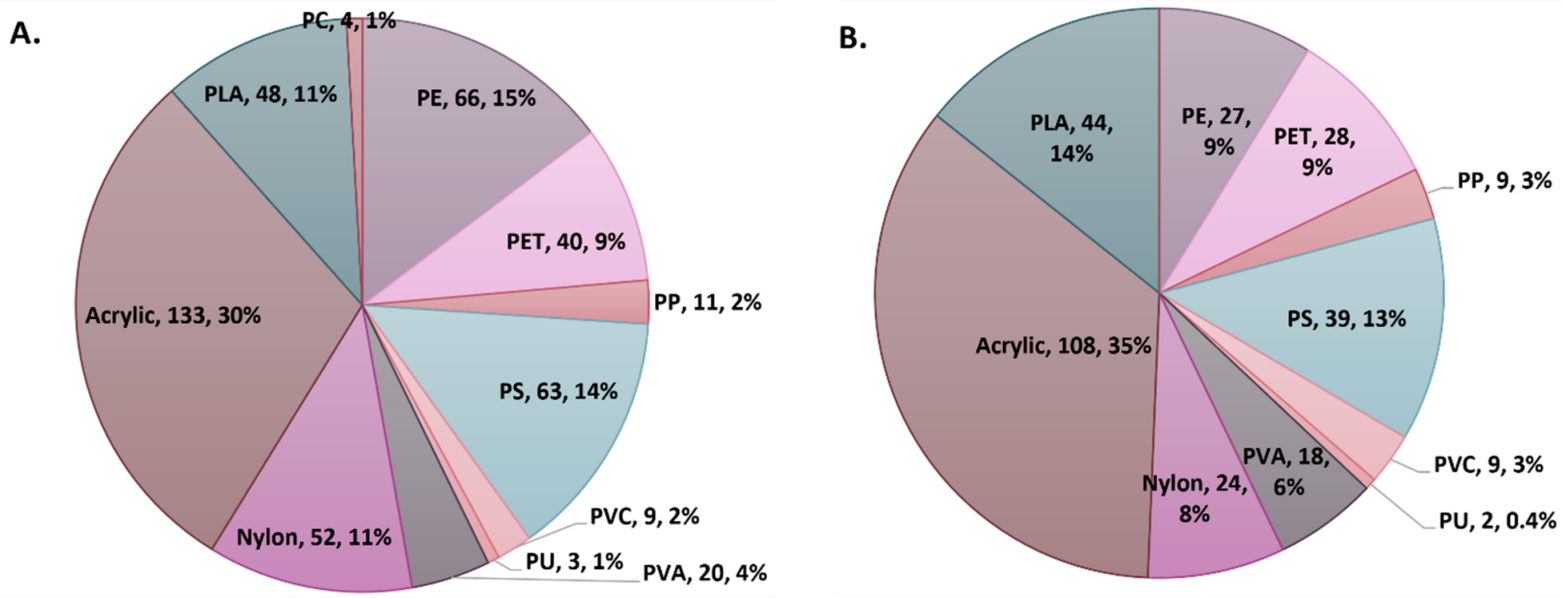
Percentage and number of identified MPs of different polymers for all of the positively identified MP particles in this study, grouped as (A) fibers and (B) fragments.

According to a Kruskal–Wallis test, no significant difference in MP sizes among the different sampling frequencies was found. The average MP size of fragments for all samples was 219 ± 228 μm, with a range between 65 and 2846 μm. The average length of fibers was 1287 ± 1111 μm with a range between 119 and 4839 μm for fibers (Supporting Information S8, Microplastic size distribution). From the particles picked for chemical identification (*n*_target_ = 729), 88.8% were confirmed as plastics. For identified fibers, acrylic represented the dominant polymer category across the sampling regimens, followed by nylon, polylactic acid (PLA), polyethylene (PE), and polystyrene (PS) (Figure 2). For fragments, acrylic was the dominant polymer type across all sampling regimens, followed by PLA, polyethylene terephthalate (PET), PS, and PE (for a detailed breakdown, see Supporting Information S9, Microplastic polymer composition). Both acrylic and PLA have been associated with wastewater signals in the past as acrylic may be used as a flocculant in WWTPs^55−57^ and in many household detergents.^55^ It is also plausible that high acrylic counts might be related to some specific operations in the WWTP, such as pile cloth filtration, though this cannot be scrutinized further, as Barston has a proprietary system in place. PLA instead is a biopolymer that has a wide array of uses in sanitation products being perceived as “biodegradable”, such as wet wipes.^58,59^

### 3.2 Variations in Microplastic Concentrations

**Figure 3:**
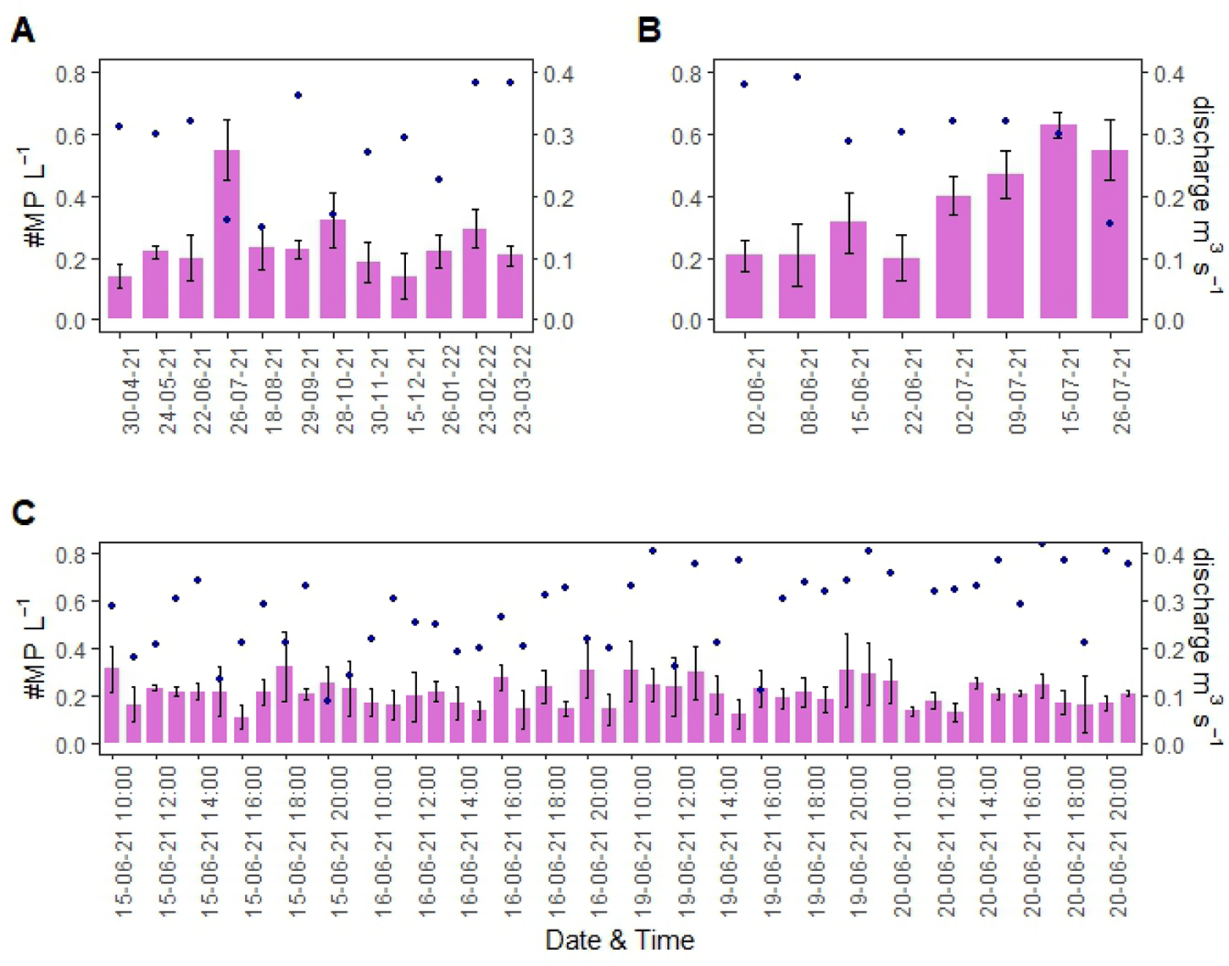
MP concentration in surface water (MPs per liter) and measured stream discharge (cubic meters per second) as obtained from (A) monthly sampling, (B) weekly sampling, and (C) hourly sampling (12 h over 4 separate days).

MP concentration data for monthly, weekly, and hourly samples are listed in Figure 3. For the year-long monthly sampling, the range of observed MP concentrations was between 0.14 and 0.55 MP L^–1^ (Figure 3A) with a mean of 0.24 ± 0.10 MP L^–1^. The largest difference in the monthly sampling was found among the months of July, April, and December (χ^2^ = 22.386; *p* = 0.021). For the weekly samples, MP concentrations ranged between 0.20 and 0.63 MP L^–1^ (Figure 3B), with a mean of 0.37 ± 0.15 MP L^–1^. A small MP concentration increase was observed between June and July, with the highest mean MP concentration recorded on July 15, 2021. On that day, MP concentrations were significantly higher than for hourly concentration data from June 2, 8, and 22 and July 2 (χ^2^ = 19.2; *p* = 0.007).

With the sampling frequency increasing to hourly sampling, high variability was observed between the days and hours sampled. When the total daily mean concentrations were considered [0.23 ± 0.06 (June 15), 0.20 ± 0.05 (June 16), 0.24 ± 0.05 (June 19), and 0.20 ± 0.04 MP L^–1^ (June 20)], no significant difference among the 4 days (χ^2^ = 4.77; *p* = 0.189) could be observed. The mean for the 4 days was 0.22 ± 0.05 MP L^–1^. According to a *t* test, there was also no significant difference between the MP concentrations on weekdays and weekend days [*t*(142) = −0.399; *p* = 0.689]. The difference in hourly MP concentrations (based on three replicates) and daily mean concentrations was assessed and showed that on June 15, only 1 h provided concentrations that were significantly different from the daily mean (4 p.m.; *p* = 0.018; effect size = 0.707). On June 16 and 19, no significant difference was observed. For June 20, there was one significantly different sample (2 p.m.; *p* = 0.040; effect size = 0.707) from the daily mean.

To address any potential broad seasonal trends, the 12-month period was divided into four meteorological seasons: spring, March–May; summer, June–August; fall, September–November; winter, December–February. However, no significant difference among the four seasons [spring (0.19 ± 0.04), summer (0.33 ± 0.17), fall (0.24 ± 0.08), and winter (0.22 ± 0.08) (χ^2^ = 4.943; df = 3; *p* = 0.176)] could be observed.

### 3.3 Variations in Stream Discharge

In this study, the stream discharge downstream of the outflow of the WWTP was characterized by high temporal variability (Supporting Information S1, Hydrograph for discharge). The discharge from the WWTP varied in a broad diurnal cycle, peaking twice a day at approximately 9:30 a.m. and 9:30 p.m. (Supporting Information S1, Hydrograph for discharge). However, within this daily release cycle, the variability between the minimum and maximum discharge was high even within only 15 min, varying between 30 and 200 L s^–1^, and underlining the large fluctuations in WWTP outflow.

**Figure 4:**
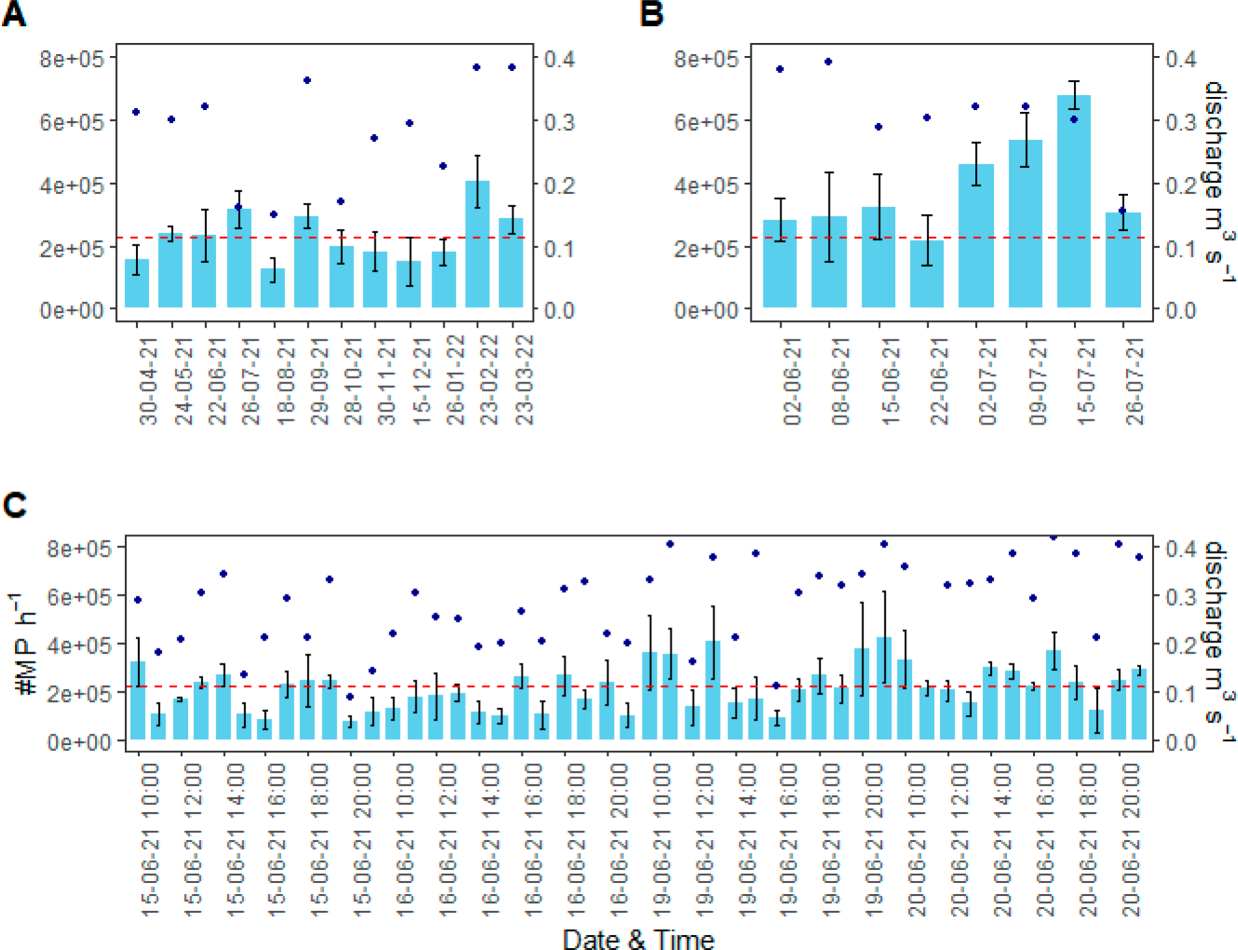
Measured stream discharge (cubic meters per second) indicated by blue dots and calculated MP loads (MP per hour) indicated by bars, based on (A) monthly samples (collected in the last two weeks of each month), (B) weekly samples (June 2, 8, 15, and 22 and July 2, 9, 15, and 26, 2021), and (C) hourly sampling over 12 h, with two weekdays (Tuesday and Wednesday, June 15 and 16, 2021, respectively) and two weekend days (Saturday and Sunday, June 19 and 20, 2021, respectively). The dashed line indicates the annual mean MP load (224 900 MPs h^–1^) calculated on the basis of monthly sampling, and the error bars show the standard deviation of the triplicate water samples collected during microplastic sampling.

Stream discharge measured along with the monthly microplastic sampling varied between 0.15 m^3^ s^–1^ (August 18, 2021) and 0.38 m^3^ s^–1^ (March 23, 2022), revealing no clear pattern (Figure 4A). The stream discharge measured along with the weekly microplastic sampling ranged from 0.16 m^3^ s^–1^ (July 26, 2021) to 0.39 m^3^ s^–1^ (June 8, 2021). The hourly measurements revealed high variability in stream discharge, with rapid changes of as much as 0.20 m^3^ s^–1^ within just 1 h (Figure 4C). *t* test results suggest that the observed higher discharge during the two weekend days as compared to the two weekdays was statistically significant [*t*(46) = −4.32; *p* < 0.001]. For further details, see Supporting Information S2 (Metadata for sampling events).

### 3.4 Variations in MP Loading

The average MP loads (MPs per hour) determined using the different sampling frequencies were statistically not significantly different (χ^2^ = 66.874; *p* = 0.378). However, the observed range between the minimum and maximum MP load varied with sampling frequency, revealing that high variability is present within MP loads (Figure 4). The average MP load identified on the basis of the monthly sampling campaigns was 224 900 MPs h^–1^ with a range from 124 200 (August) and 384 000 MPs h^–1^ (February) for different months. Some of the largest identified MP loads coincided with the highest stream discharges, although the month of July (July 26, 2021) was characterized by a high load despite a relatively low stream discharge (0.16 m^3^ s^–1^) (Figure 4) and was rather driven by MP concentration, which was the highest (0.55 MP L^–1^) found in the monthly samples.

The average MP load determined by the weekly sampling was 387 500 MPs h^–1^ with a range from 229 400 to 675 700 MPs h^–1^. Here, the range identified by weekly sampling was similar to that of the monthly sampling (factor of 2.9 vs factor of 3.1). The weekly sampling revealed an increasing trend of MP loads for the first three weeks of July (Figure 4), with values clearly above the annual average (Figure 4). Hourly sampling revealed an even larger fluctuation in MP loads, with a 1 order of magnitude difference being observed within a single day (June 15) (Figure 4). For the weekdays, the range of MP loads on June 15 varied between 85 600 and 323 300 MPs h^–1^ with an average of 186 600 MPs h^–1^ with the lowest MP load estimate occurring at the same time as the lowest MP concentration (4 p.m.) and the highest load at the time of the second highest MP concentration recorded for the day (10 a.m.). For June 16, the range of MP loads was between 101 800 and 273 200 MPs h^–1^ with an average of 175 700 MPs h^–1^. The lowest MP load was found for the 3 p.m. sample, which showed a MP concentration similar to that of samples collected at 9 a.m., 5 p.m., and 7 p.m., while the highest load occurred at the time of the second highest MP concentration at 4 p.m. For the weekend, the range of the MP loads on June 19 was between 93 300 and 425 900 MPs h^–1^, with an average load of 267 200 MPs h^–1^. The lowest MP load was estimated for the sample taken at 4 p.m., which had the median MP concentration (the sixth lowest/highest), while the highest MP load was quantified for the sample taken at 9 a.m., which showed the fourth highest MP concentration. On June 20, the MP load ranged between 127 000 and 382 600 MPs h^–1^ with an average of 251 200 MPs h^–1^. The lowest MP load was found at 7 p.m., which had the third lowest MP concentration, and the highest load was estimated for the sample at 5 p.m., which exhibited the third highest MP concentration. The hourly sampling also suggested that the weekdays (June 15 and 16) were characterized by significantly lower MP loads (average of 186 600 MPs h^–1^ on June 15 and 175 700 MPs h^–1^ on June 16) than weekend days (267 200 MPs h^–1^ on June 19 and 251 200 MPs h^–1^ on June 20) [*t*(46) = 3.212; *p* = 0.002].

### 3.5 MP Relationships with Stream Hydrological Parameters

MP concentrations were compared to stream hydrological parameters to assess relationships that indicate source or transport controls of the observed MP dynamics. The stream electrical conductivity (EC, measured in millisiemens per centimeter) as a potential indicator of the concentration of released wastewater was negatively correlated with the stream discharge at the times of sampling (*R*_s_ = −0.35; *p* < 0.001). However, EC was not correlated with MP concentration (*R*_s_ = 0.01; *p* = 0.862). The stream discharge was not correlated with the observed MP concentration [*R*_s_ = −0.26; *p* = 0.418 (Supporting Information S10, Stream discharge and microplastic concentration)] or recorded MP sizes (*R*_s_ = 0.10; *p* < 0.001). Flow velocity did not correlate with MP concentration in the stream either (*R*_s_ = −0.10; *p* = 0.555) or with MP size spectra (*R*_s_ = 0.08; *p* = 0.002).

## 4 Discussion

### 4.1 Temporal Variability in Stream Discharge Is an Important Driver of Downstream MP Transport

Significant temporal variability in stream discharge has been observed for a multitude of riverine settings.^60^ This temporal variability can be linked to seasonal weather patterns (e.g., snowmelt), event-based weather extremes (e.g., floods), or human water use (e.g., for agriculture, water supply, and energy production).^61−63^ Although MP downstream transport patterns seem in many settings to be closely related to stream discharge variations,^64^ so far, comparatively few studies have considered the temporal aspects of these variations, specifically in isolation from the spatial variations.^65,66^ This arises from a general lack of time-dependent MP concentration data in different stream settings. Additionally, riverine MP concentration data are hard to interpret. MP concentration time series in many settings represent a superposition of signals from point source and non-point source data, where the various sources often cannot be easily isolated. Additionally, as MPs are particles, these concentration time series will also be impacted by the relevant particle transport mechanisms (e.g., gravitational settling, burial, suspended and bedload transport, hyporheic exchange, etc.) upstream of the sampling point. These transport mechanisms differ for different MPs depending on their physical and chemical characteristics (e.g., shape, density, size, and surface patterns) as well as due to changing environmental factors.^67−69^ Other aspects such as MP mixing behavior in the water column and degradation due physical and biochemical processes upstream of a sampling point also impact the concentration time series data.

Despite these difficulties, the temporal variability in stream discharge seems to be one major driver in downstream MP transport as, for example, shown in a recent study on the River Weser, Germany.^65^ The results obtained in our study, however, highlight the impact that high temporal streamflow variability and MP concentration in the water column can have on subsequent MP load calculations. For example, for hourly samples, the highest MP loads never coincided with the highest MP concentrations, and only on October 19, 2021, did the highest MP load coincide with the highest recorded stream discharge. This underlines the importance of adequately covering representative MP sampling time scales for different flow conditions. While stream discharge at our sampling site was highly variable due to fluctuating WWTP outflows (Figure 4 and Supporting Information S1, Hydrograph for discharge), the WWTP effluent also represented the only significant MP source at the sampling site. By combining time-varying discharge and concentration information, we were able to identify periods when changes in MP loads were mostly driven by a change in discharge (e.g., Figure 4B, July 15 vs July 26, 2021) or those periods when significant MP load changes occurred despite rather minor changes in discharge (e.g., Figure 4B, June 15 to July 15, 2021). Those latter periods thus suggest a significant change in the MP concentration in the effluent of the WWTP. As the WWTP is both the only significant source of water and plastic pollution at the downstream sampling point, a significant change in MP concentration in its effluent suggests a change in the WWTP’s removal efficiency or the MP concentration within the influent. A reduced removal efficiency could be linked to the release of at least partially untreated wastewater during times of high rainfall. However, according to the Meteorological Office (MET), for the nearby Coleshill station (NGR = 4211E 2869N, 52.48, −1.689) no rainfall event had occurred during the specific days of sampling or within the 2 days prior to these dates and further operational data from the WWTP operator were not available for those dates. As such, a significant increase in MP influent concentration to the WWTP is the more likely alternative. Determining which factors might impact this could be challenging but require further research.

The annual average MP concentrations obtained in our study (0.24 ± 0.10 MP L^–1^, >63 μm) were slightly lower than those reported for other WWTP effluent-influenced sites in studies with a similar limit of detection [e.g., 0.59 ± 0.22 MP L^–1^ (>50 μm),^70^ 2.5 ± 0.3 MPs L^–1^ (>63 μm)^71^]. Most of these differences are likely related to specific WWTP removal efficiencies, which have been reported to vary due to differences in design and operation.^34,72^ Another potential factor explaining the differences to previous studies could be that the sampling location in this study is ∼1 km downstream from the outflow point, contrary to previous studies that sampled directly at the source of the outflow. While no significant additional MP sources were found in our stream reach, which is embedded in a rural setting, MP downstream transport and potential accumulation could already have been affected by the previously discussed MP transport mechanisms causing MP concentrations to decrease in the water column along the stream reach.^64,73^

### 4.2 The Range of MP Loads Is Important for Characterizing Downstream Export Dynamics

WWTP outflow is often considered a continuous “hot spot” for MP pollution at the local scale,^74,75^ and typically, annual MP load estimates are based on multiplying the average annual discharge with observed MP concentrations at a given sampling time (e.g., (32,76,77), and (78)). However, as shown in Figure 3, MP concentrations at our sampling site varied between 0.14 and 0.63 MP L^–1^ (a factor of 4.5), which coupled with changes in recorded discharge amounted to differences of ≤77% in hourly MP loads, indicating the need for load calculations to be based on more than just one-point-in-time concentration data. This seems especially advisable in field settings similar to the one discussed here where changes in MP load can at times be controlled by the change in discharge or WWTP effluent concentration, as discussed in section 4.1. For our sampling site, both of these parameters were also highly variable (Figures 3 and 4) and uncorrelated. The calculated MP loads ranged from 85 600 to 675 700 MPs h^–1^ for the different sampling regimes (section 3.4), and it was found that MP loads varied across all considered time scales (from hourly to annual). For example, hourly sampling suggests that depending on the time of the day when the sampling occurred, a snapshot (one-point-in-time) sampling approach could result in over- or underestimation of MP loads by ≤3.8 billion particles per year (a change of ≤200%). This high variability in MP loads between individual sampling events encountered at our site suggests that taking a snapshot sampling approach to estimate annual MP loads would come with large uncertainties. The rapid changes in MP loads even throughout 1 day (Figure 4C) suggest that MP release patterns of this WWTP and certain point sources in general might vary considerably over time and thus should be studied in detail before assumptions about average MP loads can be made. This becomes even more important in scenarios in which the respective point source represents a major MP source for a downstream river network.

The WWTP effluent composition might significantly and frequently change with treatment efficiency, influent MP load, and WWTP design. In many cases, WWTPs release effluent in accordance with local governmental regulations and depending on the physical and chemical conditions in the receiving stream. This might result in a near-pulse release pattern of MPs with the WWTP effluent, which can have wider implications across the river network as these MP-loaded “pulses” travel downstream with often unknown consequences for downstream ecosystem and biotic health. Previous work has suggested that moving from areas of high to low flow can lead to deposition of MPs to the sediment (Tibbets et al., 2018)^100^, so these pulses might also lead to accumulation of MP at lower-energy sites downstream. As such, our study emphasizes the need for a sampling approach that covers multiple time periods to capture streamflow and MP concentration dynamics more systematically. The resulting MP load calculations will be more robust, and MP transport dynamics through the downstream river network can then be predicted with higher certainty.

### 4.3 Sampling at Different Intervals Is Required to Better Characterize MP Fate and Transport

Within the investigated stream reach, the high variability in both discharge and MP concentrations resulted in variable MP loads at our sampling point. For river reaches with similarly contributing point sources, this variability should be properly captured using a multiple-time scale sampling approach, to enable stakeholders and policy makers to more adequately design river management or pollution prevention plans and minimize negative consequences to the receiving ecosystem downstream.

The results reveal that for our study site, which is dominated by a single MP point source, and most likely for other sites with similar characteristics, monthly or even weekly sampling regimes may be insufficient to capture a representative range of MP concentrations. For example, MP concentrations appeared to be highest in the summer (July 2021) when considering only monthly sampling intervals (Figure 3A). Analysis of weekly samples indicated a slow increase in MP concentration from June toward the end of July (Figure 3B), However, hourly sampling over subsequent days (Figure 3C) suggests that this trend could have been observed by pure chance. Despite the sampling taking place consistently between 9 and 11 a.m., the hourly data suggest that MP concentrations at our sampling site could fluctuate by as much as 5-fold between samples collected only 1 h apart. This highlights the risk of misinterpretation when generalizing from limited seasonal or monthly sampling data regardless of the consistent sampling time and procedure, let alone generalizing from a single snapshot. A fluctuation in MP concentrations by 1 order of magnitude in WWTP effluent has been reported previously,^79,80^ while considerable short-term variations in pollutant loads in WWTP have also been described for pharmaceuticals, personal care products, and other emerging contaminants.^46,81,82^ The coefficients of variation (for details, see Supporting Information S11, Assessing the coefficient of variation for hourly microplastic concentrations) suggest that for our site five hourly samples per day would sufficiently capture the variability in MP concentrations on any given day with additional sampling required to observe possible longer-term trends.

For MP characteristics such as shape, size distribution, color, or polymer type, none of these characteristics varied significantly with sampling frequency, as the site was dominated by single point source, where no significant changes in output would be anticipated due to similar inputs and removal of certain MP sizes, some considerable differences in a few of the samples were observed. For example, fragments are the most dominant shape for all samples but those collected at the end of July 2021 (Supporting Information S7, Microplastic morphology distribution), which see fibers as the dominant shape suggesting a change in either MP influent composition or WWTP treatment efficiency. There is a growing body of evidence that some biota such as certain fish species preferentially take up fibers of specific colors, potentially confusing these with their prey items.^13,83,84^ As such, these types of changes can have a direct impact on the biota downstream from the sampling site. Similarly, when looking at the MP size range (Supporting Information S8, Microplastic size distribution), we can observe that the percentage of particles in the size range of 126–250 μm was ∼50% for all sampling dates. This could have a profound impact on downstream species that may be able to take up these smaller particles more readily. Consequentially, exposure calculations need to account for a change in the MP load as well as in MP characteristics over time. For the latter, again, it might be important to study the expected range of characteristics by using a multiple-time scale sampling approach rather than just relying on results from snapshot sampling.

### 4.4 Implications and Future Research

This study showed that at the sampling site with WWTP effluent as the only significant source of water and MPs, the measured stream discharge, MP concentration, and subsequent load estimates as well as MP characteristics varied considerably with time. To better capture this variability, more conclusive information can be gained by increasing the sampling frequency beyond occasional snapshot sampling. While WWTPs have previously been termed important and steady sources of MPs,^32,33,74,75^ our results strongly suggest that at least for river reaches similar to the one investigated here repeated sampling at multiple time scales seems beneficial for more robustly determining MP release patterns and downstream transport. Many reaches often receive MPs from multiple point and diffuse sources, and the contribution of an individual source to total MP concentrations and loads usually remains hidden because of the superposition of these different signals as well as the impact of dilution from upstream waters. However, as even slight changes in MP loads, concentrations, and characteristics over time can have a profound impact on downstream ecosystems, a robust system characterization is a prerequisite to understanding and quantifying risks to which these downstream ecosystems are exposed and to designing appropriate cleanup measures or management plans where required. As such, monitoring MP fate and transport at multiple time scales or where possible quasi-continuously as is done for nutrients or other dissolved contaminants^85^ could be beneficial for a multitude of river settings with variable flow conditions. Such monitoring could also help stakeholders identify times of increased MP release upstream of a site of water abstraction to minimize carryover effects of MPs to the wider catchment or adjacent catchments.^7^ A better temporal resolution of MP release patterns near major point sources can also help to improve MP transport and fate models.

Using a multiple-time scale sampling approach, as discussed in this study, might not be feasible in all instances as we had invested considerable resources in sampling and analysis. We recommend that to obtain a better understanding of the system dynamics in those systems with major point sources such as WWTPs, a premonitoring campaign/pilot study be conducted, which could include measurements of discharge, velocity, and MP concentration in the source effluent or immediately downstream of the site. Measurements could take place over a week on a daily or even hourly basis to understand their variability and thus to determine the optimal and minimum sampling frequency needed to provide a reasonable representation of the MP concentration. This could lead to better planning for the overall study, thus maximizing the effectiveness of subsequent sampling campaigns and helping to avoid the collection of nonrepresentative samples, as well as aiding in local exposure assessments based on the resulting data. To this end, we hope that future research will focus on the improvement of *in situ* sensors that can help to continuously monitor particle load and robustly discriminate between nonplastic and MP particles in the water column or monitor a proxy water quality parameter that could be related to MP concentration (such as total suspended solids).^86,87^ Those sensors could then be incorporated into existing water quality monitoring networks to allow for near-real-time data on MP loads.

## 5 Conclusion

While WWTPs have been quoted to be a steady point source of the release of MPs into rivers, this study suggests that a snapshot (one-point-in-time) sampling approach could result in over- or underestimation of MP loads by ≤3.8 billion particles per year, as MP concentrations and loads can vary significantly on an hourly basis. The importance of premonitoring campaign/pilot studies is highlighted, as the proper characterization of study sites will lead to a better understanding of the patterns of release of MPs from WWTPs into rivers, which will aid future monitoring and mitigation activities by regional stakeholders and/or water companies.

## Supporting Information Available

Additional experimental details,equipment configurations and figures showing microplastic morphology, color, size, and polymer composition (PDF)
