# Supplementary material for: Snapshot Sampling May Not Be Enough to Obtain Robust Estimates for Riverine Microplastic Loads
Source: ACS ES T Water. 2024 Apr 12;4(5):2309–19. doi: 10.1021/acsestwater.4c00176 (PMC11091885; doi:10.1021/acsestwater.4c00176)
Supplement: Supplementary file 1 — ew4c00176_si_001.pdf [file ew4c00176_si_001.pdf]

Snap-shot sampling may not be enough to obtain robust estimates for riverine microplastic loads

Anna Kukkola[a*], Uwe Schneidewind[a], Lee Haverson[a], Liam Kelleher[a,c], Jennifer D. Drummond[a], Gregory Sambrook Smith[a], Iseult Lynch[a,c] and Stefan Krause[a,b,c]

[a] School of Geography, Earth and Environmental Sciences, University of Birmingham, Edgbaston, Birmingham B15 2TT, United Kingdom

[b] LEHNA- Laboratoire d'ecologie des hydrosystemes naturels et anthropises, University of Lyon, Darwin C & Forel, 3-6 Rue Raphaël Dubois, 69622 Villeurbanne, France.

[c] Institute of Global Innovation, University of Birmingham B15 2SA, Birmingham. United Kingdom

*Email: a.t.kukkola@bham.ac.uk

Supporting Information

Number of pages: 18

Number of figures: 10

Number of tables: 3

S1: Hydrograph for discharge

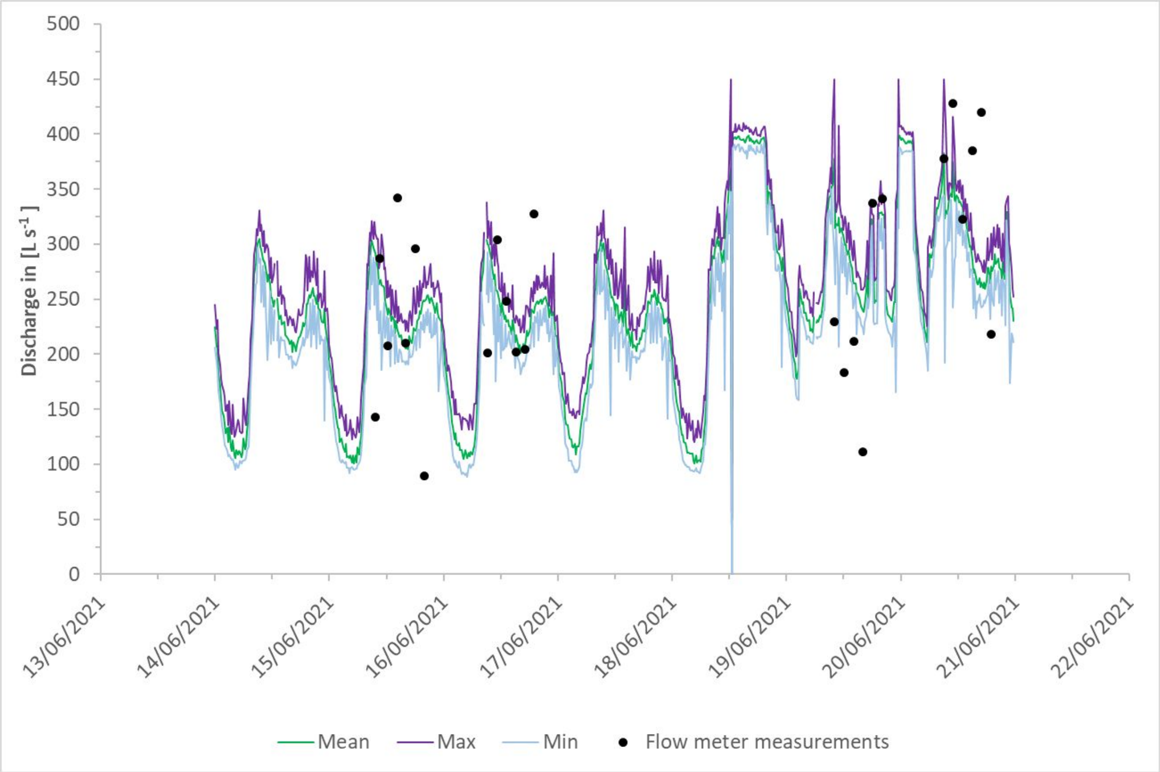

Figure S1. Discharge reported by Barston WWTP for the period of 14th June – 20th June 2021. Mean flow (green), maximum flow (purple) and minimum flow (blue) reported in 15 min intervals. Black dots represent total discharge values at our sampling location, determined by flowmeter measurements. The offset between Barston WWTP and the sampling site (about 1 km downstream) is roughly 1.5-3 hours depending on flow velocities. It can be seen that most of the water at the downstream sampling point originates from the WWTP.

S2: Metadata for sampling events.

Table S1: Details on average (n=3) microplastic (MP) concentrations [MP L^{-1}] obtained from the triplicate samples, stream discharge [m^3 s^{-1}] and MP loads [MP h^{-1}].

Sampling Date	***Sampling time (hour)***	***Stream discharge***	***Average MP con.***	***MP load [MP h^{-1}]***	***EC [mS/cm]***

		$[m^3 s^{-1}]$	$[MP m^3]$		
30/04/2021	10	0.31	140.0	156,719	0.604
24/05/2021	10	0.30	220.0	237,579	0.488
22/06/2021	10 * from weekly	0.32	200.0	229,409	0.609
26/07/2021	9 * from weekly	0.16	546.7	305,136	0.596
18/08/2021	9	0.15	233.3	124,183	0.620
29/09/2021	10	0.36	226.7	294,381	0.425
28/10/2021	9	0.17	320.0	194,951	NA
30/11/2021	10	0.27	186.7	178,339	NA
15/12/2021	11	0.29	140.0	147,927	NA
26/01/2022	11	0.21	220.0	162,466	NA
23/02/2022	11	0.36	293.3	384,076	NA
23/03/2022	10	0.38	206.7	283,596	NA
02/06/2021	10	0.38	78.4	282,092	0.595
08/06/2021	10	0.39	80.9	291,260	0.580
15/06/2021	10 *from hourly	0.30	313.3	337,838	0.605
22/06/2021	10	0.32	63.7	229,409	0.609
02/07/2021	10	0.32	127.4	458,819	0.604
09/07/2021	10	0.32	148.5	534,581	0.567
15/07/2021	10	0.30	187.7	675,677	0.589
26/07/2021	9	0.16	84.8	305,136	0.596
15/06/2021	9	0.14	233.3	120,312	0.603
15/06/2021	10	0.30	313.3	337,838	0.605
15/06/2021	11	0.18	166.7	108,936	0.610
15/06/2021	12	0.21	233.3	174,185	0.592
15/06/2021	13	0.30	220.0	238,941	0.588
15/06/2021	14	0.34	220.0	270,924	0.579
15/06/2021	15	0.14	220.0	107,201	0.597

15/06/2021	16	0.21	113.3	85,691	0.608
15/06/2021	17	0.29	220.0	231,622	0.643
15/06/2021	18	0.21	326.7	246,993	0.627
15/06/2021	19	0.33	206.7	245,086	0.627
15/06/2021	20	0.09	266.7	85,922	0.611
16/06/2021	9	0.20	146.7	106,182	0.595
16/06/2021	10	0.22	180.0	141,604	0.592
16/06/2021	11	0.30	173.3	189,925	0.585
16/06/2021	12	0.26	206.7	190,083	0.583
16/06/2021	13	0.25	220.0	196,441	0.585
16/06/2021	14	0.19	173.3	119,060	0.587
16/06/2021	15	0.20	140.0	101,806	0.598
16/06/2021	16	0.26	286.7	273,200	0.615
16/06/2021	17	0.20	146.7	107,907	0.625
16/06/2021	18	0.31	240.0	268,647	0.616
16/06/2021	19	0.33	146.7	172,830	0.604
16/06/2021	20	0.22	306.7	241,250	0.605
19/06/2021	9	0.40	141.2	205,067	0.491
19/06/2021	10	0.33	127.5	151,182	0.506
19/06/2021	11	0.40	141.2	205,067	0.485
19/06/2021	12	0.16	81.8	47,283	0.486
19/06/2021	13	0.38	136.4	184,433	0.489
19/06/2021	14	0.21	101.7	77,535	0.486
19/06/2021	15	0.38	138.0	191,258	0.506
19/06/2021	16	0.11	66.1	26,438	0.538
19/06/2021	17	0.30	121.6	132,017	0.533
19/06/2021	18	0.34	141.4	171,757	0.542
19/06/2021	19	0.32	125.6	144,723	0.547

19/06/2021	20	0.34	148.8	182,938	0.550
20/06/2021	9	0.38	151.4	205,789	0.479
20/06/2021	10	0.36	133.0	170,951	0.455
20/06/2021	11	0.43	162.5	250,130	0.434
20/06/2021	12	0.32	125.6	144,723	0.445
20/06/2021	13	0.32	130.4	151,323	0.445
20/06/2021	14	0.33	127.5	151,182	0.443
20/06/2021	15	0.38	152.6	211,342	0.473
20/06/2021	16	0.29	119.5	125,777	0.491
20/06/2021	17	0.42	157.8	238,319	0.503
20/06/2021	18	0.38	138.0	191,258	0.509
20/06/2021	19	0.21	98.5	75,071	0.513
20/06/2021	20	0.40	141.2	205,067	0.514

S3: Sample Preparation, digestion, and staining

The samples were extracted by pouring the contents of the 20 mL vials onto a 63 µm sieve (nylon mesh), after which the vials were rinsed several times with DI-water into the sieve ensuring that all contents were removed. Chemical wet peroxide oxidation was used to digest organic matter (OM) at a ratio of 1:10 [1]; 20 mL 30% H_2O_2 was used to backwash the contents of the sieve into 250 mL beakers, before adding 2 mL Fe^{2+} (aq) (0.05 M) as a catalyst. Samples were covered with loose aluminium foil lids, allowing for any heat/gas to escape, while avoiding airborne contamination, and then left for at least 24 hours at room temperature to allow degradation of the OM.

For Nile Red staining, the oxidized samples were first decanted through clean 63 µm sieves and then backwashed into beakers with DI-water. A stock solution of 1 mg mL^{-1} Nile Red was added to bring the sample to 5 µg mL^{-1} Nile Red and left covered on an orbital shaker (Grant-bio, PSU-20i) at 105 rpm to stain for one hour. The samples were then filtered using a glass vacuum-filtration system (Merck) onto glass-fibre GF/D filters (Whatman, diameter 47 mm, pore size 2.7 µm) and rinsed with DI-water. Yellow goggles and UV-light were used to inspect beakers and

the filtration unit to ensure all visible particles had been removed. The filters were transferred into pre-labelled clean PP Petri dishes and dried for 24 hours at 50 °C.

S4: Microscopy and spectroscopy

Each filter was observed under fluorescence mode with a Macro zoom microscope (Olympus MVX-ZB10) equipped with 1 x 0.25 N objective (MVPLAPO 1X, Olympus), U-M49002XL GFP filter cube (excitation filter: 470/40 nm, dichroic mirror: 495 nm high pass, emission filter: 525/50 nm) and 130 W U-HGLGPS mercury apo lamp light source. Samples were observed under 12% light intensity on 32x magnification with 50 ms exposure time.

A set threshold of 100 fluorescence a.u. (arbitrary units) was used to select particles of interest for further consideration. Each putative MP was also observed under bright-field and had to comply with pre-set selection criteria via an identification key [2]. Data collected at this stage consisted of longest length (size), colour (with particles distinctly stained as pink due distinctly Nile Red categorised as their own category) and morphology. For polymer identification, a minimum of 40% per sampling occasion of counted putative MP was analysed (n_{total} = 729). These particles were transferred into clean glass vials with filtered DI-water with fine tweezers before being filtered onto Whatman Anodiscs (diameter 25 mm, pore size 0.2 µm). A Renishaw InVia Qontor Raman microscope equipped with a 785 nm laser was used for polymer identification with the detailed configuration provided in Supplementary information S2 and further discussed in [3]. For spectral matching, the fingerprint region of 650-1700 cm^{-1} was assessed for prominent peaks. The spectral peaks were exported into a custom Python script (Python 3.9) (Alqrinawi, F., & Kelleher, L. (2024). Raman Analyzer (Version 1.0.0) [Computer software].https://github.com/Fuadqr/Raman-Analyzer-V1.0) and peaks were matched to reference libraries, with >70% match quality index considered as positive identification. The spectral library consisted of the SLOPP, SLOPP-E [4] and an in-house reference library (Table S2). While 88.8% of the particles were confirmed as plastics, no transformation was carried out for the final MP results, due to some organic matter being transferred unintentionally onto the Anodiscs with the suspected MPs, which can lead to added negatives that would skew the apparent accuracy of the fluorescent result [2].

For MP identification, a 5x objective was used, with slit width 65 µm, 1200l/mm, spatial resolution < 1 µm and a spectral resolution < 1cm^{-1}, laser intensity 10% of the system (approximately 15 mW), 3 accumulations of 5s exposures per MP particle. Cosmic ray removal, baseline subtraction (adaptive polynomial fitting) and smoothing (Savitsky-Golay filter with interval 10 and polynomial order 3) were applied using Spectragryph v1.2.16.1. The polymers tested for with the applied in-house library are listed in Table S2.

Table S2: The in-house polymer library with the polymers and number of samples for each type. Three particles were measured from each sample with the settings specified above.

POLYMER	NUMBER OF SAMPLES
**LDPE**	3
**LLDPE**	2
**MDPE**	2
**HDPE**	4
**ULDPE**	1
**PES**	2
**PET**	6
**PP (ALSO EXPANDED PP)**	7
**PS (ALSO EXPANDED PS)**	3
**PVC**	3
**PU**	2
**ABS**	3
**PC**	2
**PVA**	2
**PA-6**	2
**PA-66**	2
**COTTON**	4
**PMMA**	3
**RUBBER**	3
**CELLULOSE**	2
**PTFE**	2
**PLA**	2

S5 QA/QC procedures

Field background samples (n=12) were collected using pre-cleaned glass jars that were left open by the stream bank to identify any possible air deposition for the duration of the water sampling. To account for any contamination from the researchers conducting the sampling, sample handling and processing, fibre samples from personnel's clothing were taken (n=2). Researchers wore the same outer layer for each field visit throughout the project, and any fibres matching the physical properties of the respective clothing fibres were excluded from the analysis. Additionally, three procedural blanks were collected under controlled conditions to account for the sampling procedure. The researcher wore the clothes and chest waders used in the field and used the same PP-jug to filter 6 L of DI-water through the 63 µm sieve. Samples were extracted and analysed with fluorescent microscopy as described in the manuscript section 2.3.1.

All chemical solutions used were pre-filtered onto GF/D (2.7 µm pore size) glass-fibre filters. All GF/D filters were burnt off at 500 °C for 5 hours prior use. All working surfaces were cleaned prior to and after sample handling with filtered 70 % ethanol. Equipment was cleaned with detergent and rinsed with DI-water before and between usage. Glassware was used where feasible, and beakers were placed in a sonification bath (GT sonic-D9) for 10 min for further cleaning. Personnel wore 100 % cotton laboratory coats and only cotton/wool clothes were allowed in the laboratory. Nitril gloves were worn when handling samples and aluminium foil was used to cover samples during procedures. Two procedural blanks were run for each extraction batch (n_{total}=20) using DI-water and were analysed following the same criteria as for the samples.

From the twelve field background samples, three contained one clear fragment each, indicating that air deposition or potential contamination was minimal at the site. In the 20 laboratory blanks, only one fragment and one black fibre (not on the same day nor filter) were found. These were not deducted from the final counts, as no black fibres with an identical diameter and properties were encountered on the filters processed the same day and the additional laboratory blanks for the same batch indicated no contamination. Additionally, no contamination was found from the three field procedure blanks, indicating no contamination from the field equipment and it was concluded that based on this evidence, levels of contamination appear to be negligible and extremely random.

For testing the extraction efficiency of the method used here, a spiking experiment was conducted. A researcher picked fibres already identified as MP with Raman spectroscopy from Anodiscs and spiked 15 fibres (diameters 6-20 µm) into 20 mL vials with DI-water. Then a researcher added 10 particles of each of the three different polymers (PE, PLA and PS) between sizes 70 to 500 µm into the vials. These polymers were specifically chosen to represent the full spectrum from very low to high pixel brightness. The samples were extracted and analysed with fluorescence microscopy as described in manuscript section 2.3.1 and the raw results are presented in Table S3. The researcher counting the filters noted that some original particles seemed to have started to fragment or some of the particles had smaller fragments attached to them (formation of aggregates), however all were counted. Recovery rates from the spiking experiments ranged between 67 and 80 % for fibres, with smaller diameter fibres being lost most frequently (6-8 µm). The recovery for fragments ranged between 100-113 %, suggesting rather robust fragment capture. It should be noted, however, that our fibre capture seems to be a slight underestimation, with an average capture rate of 78 %. As we assume that this recovery rate remained relatively stable, no transformation was carried out, as the focus of the paper is the effect of sampling scheme, rather than total number of particles and/or fibres.

Table S3: Results of positive blank spiking experiments.

ID	Morphology	Spiked	Recovered	Recovery %
**1**	Fibre	15	12	80
**1**	Fragment	30	30	100
**2**	Fibre	15	10	67
**2**	Fragment	30	32	106
**3**	Fibre	15	13	87
**3**	Fragment	30	34	113

S6: Microplastic colours per sampling frequency

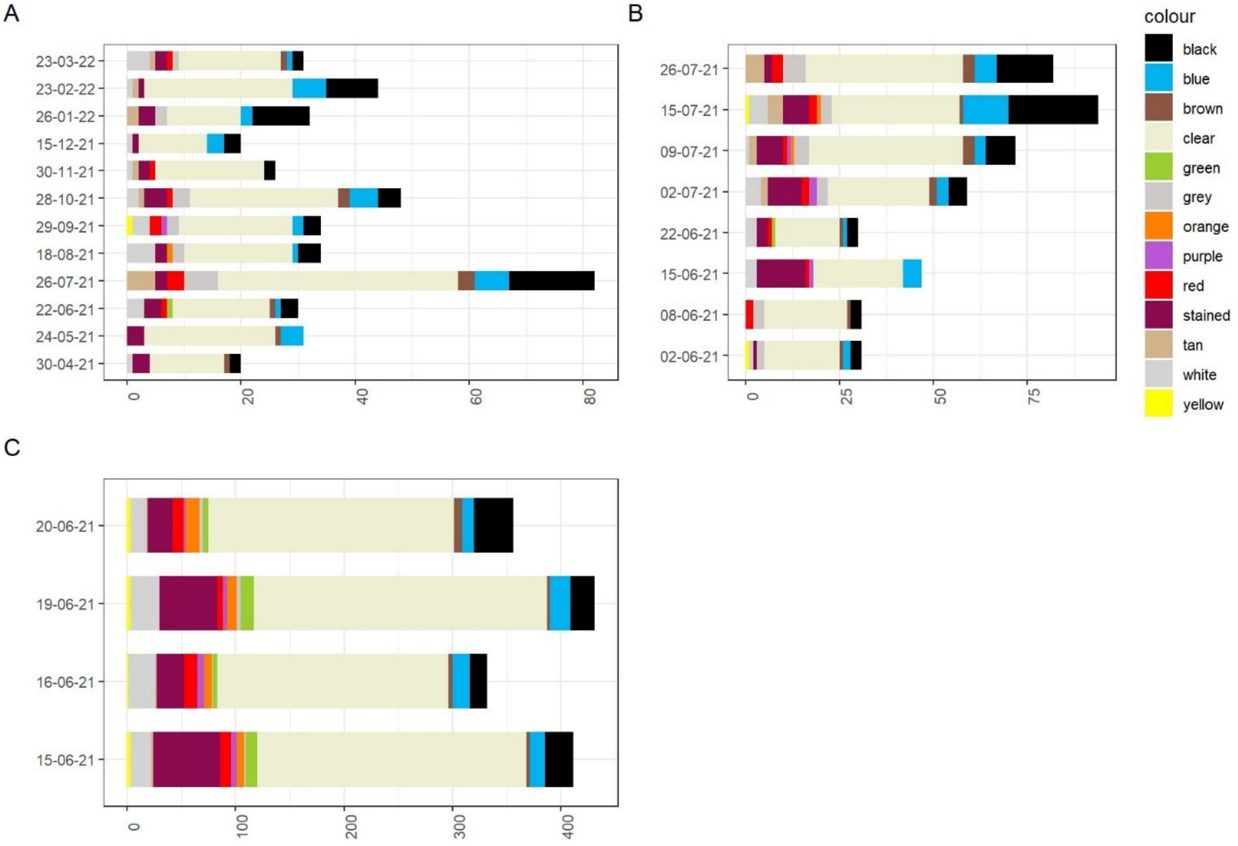

Figure S2: The respective colours of MPs extracted from the samples for: A)12-months, B) weekly and C) daily (12 x hourly samples per day over 4 days)

S7 Microplastic morphology distribution

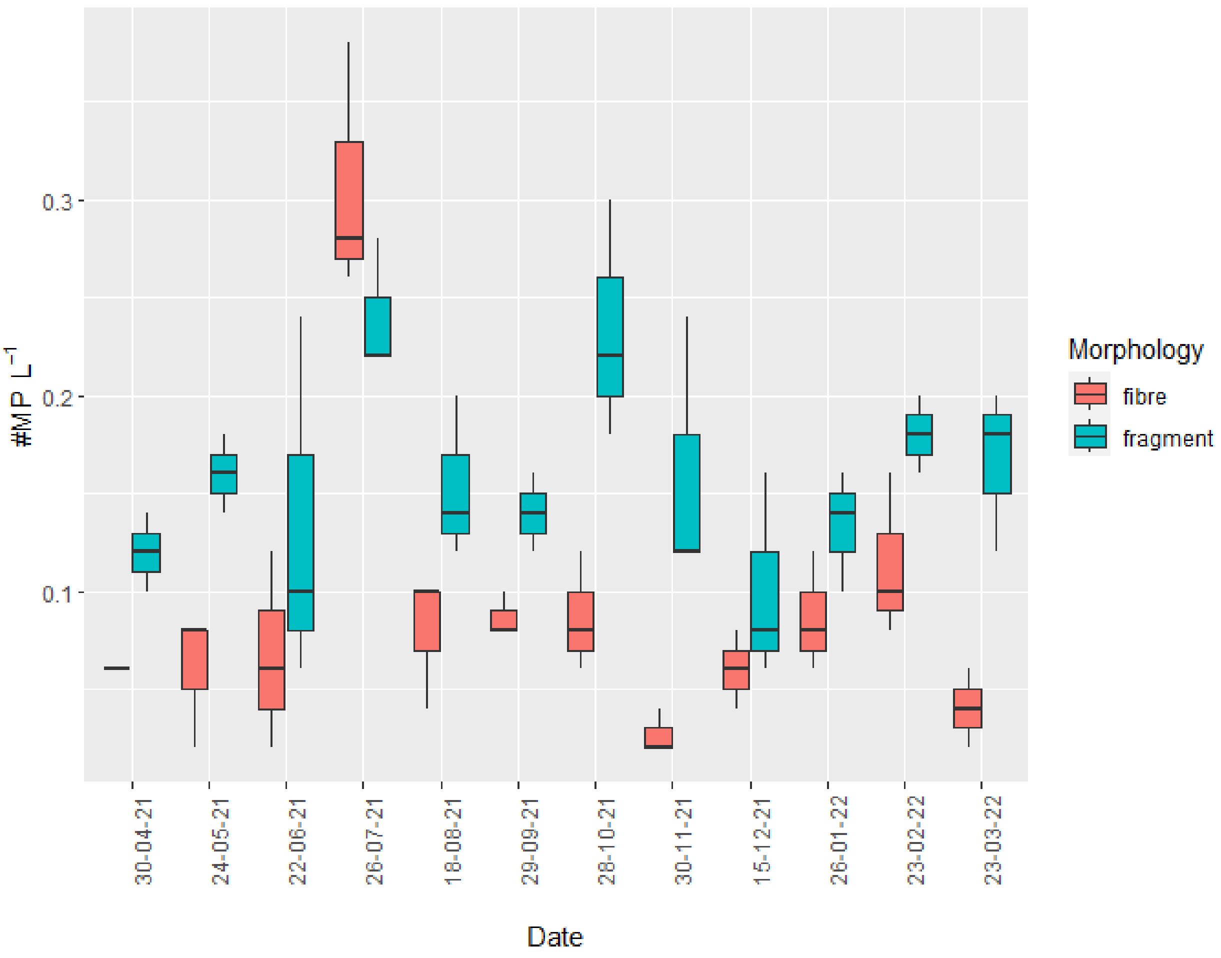

Figure S3: The relative fraction and abundance of fragments and fibres extracted from the water column samples for the 12 monthly samples.

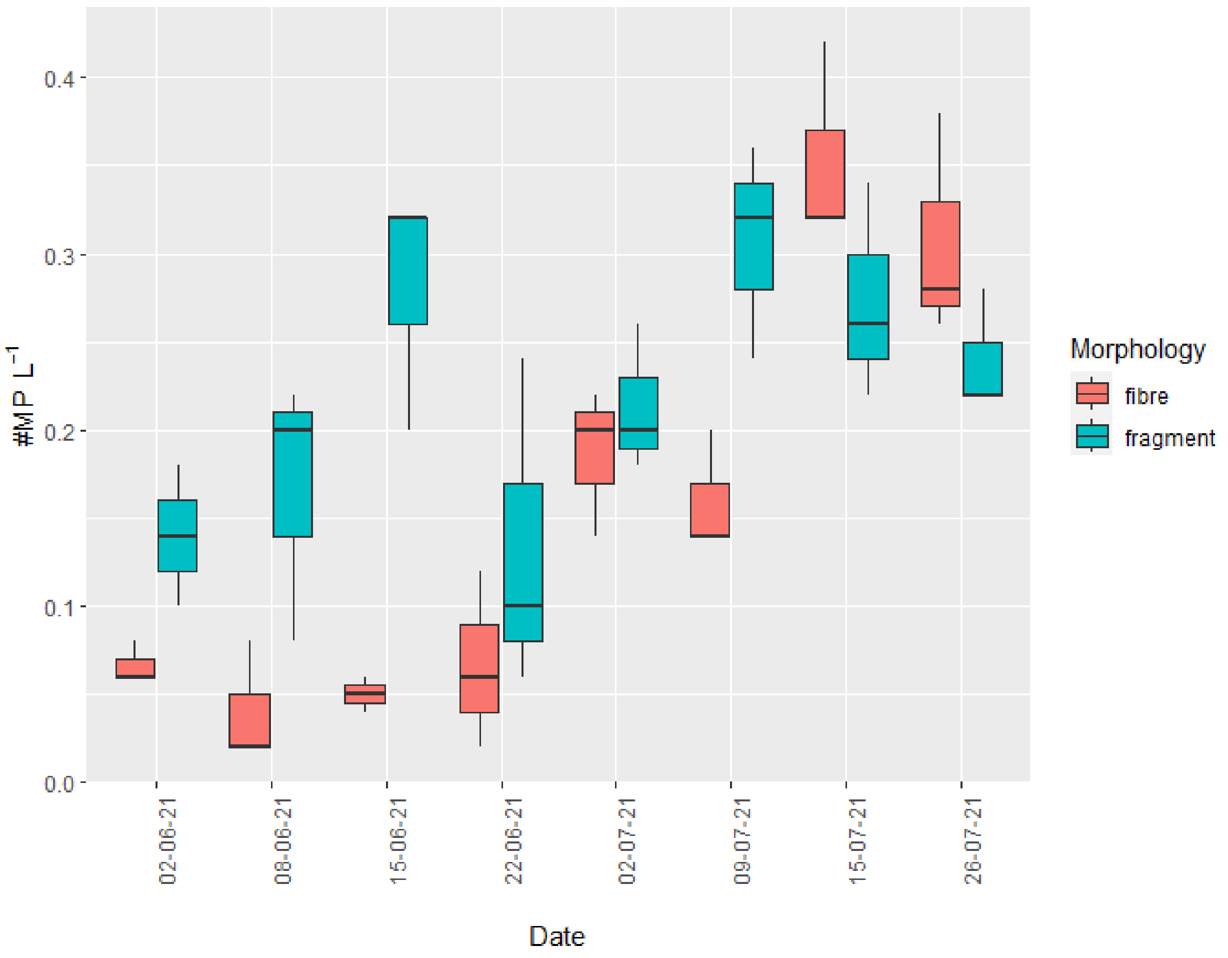

Figure S4: The relative fraction and abundance of fragments and fibres extracted from the water column samples for the weekly samples.

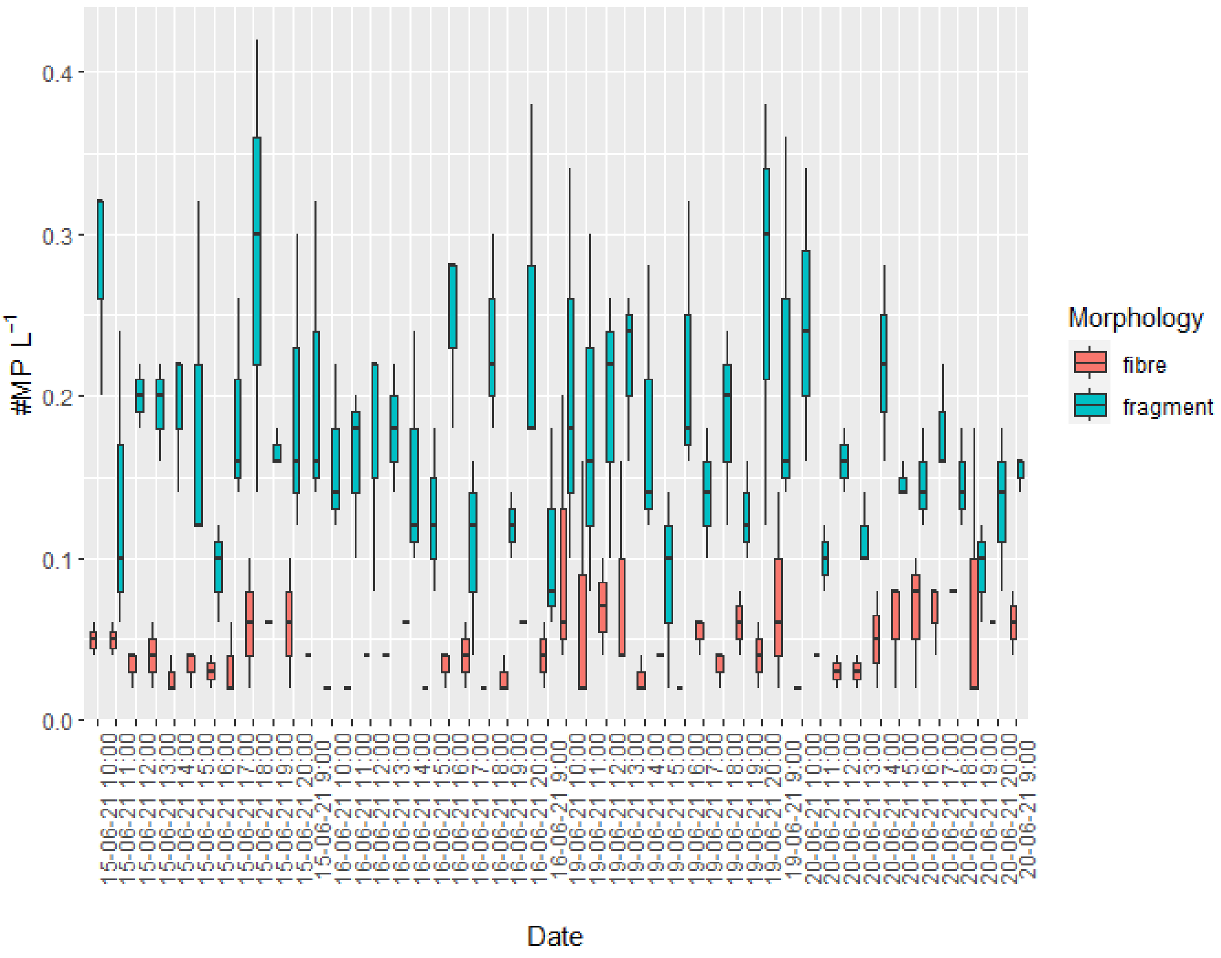

Figure S5: The relative fraction and abundance of fragments and fibres extracted from the water column samples for the hourly samples.

S8: Microplastic size distribution

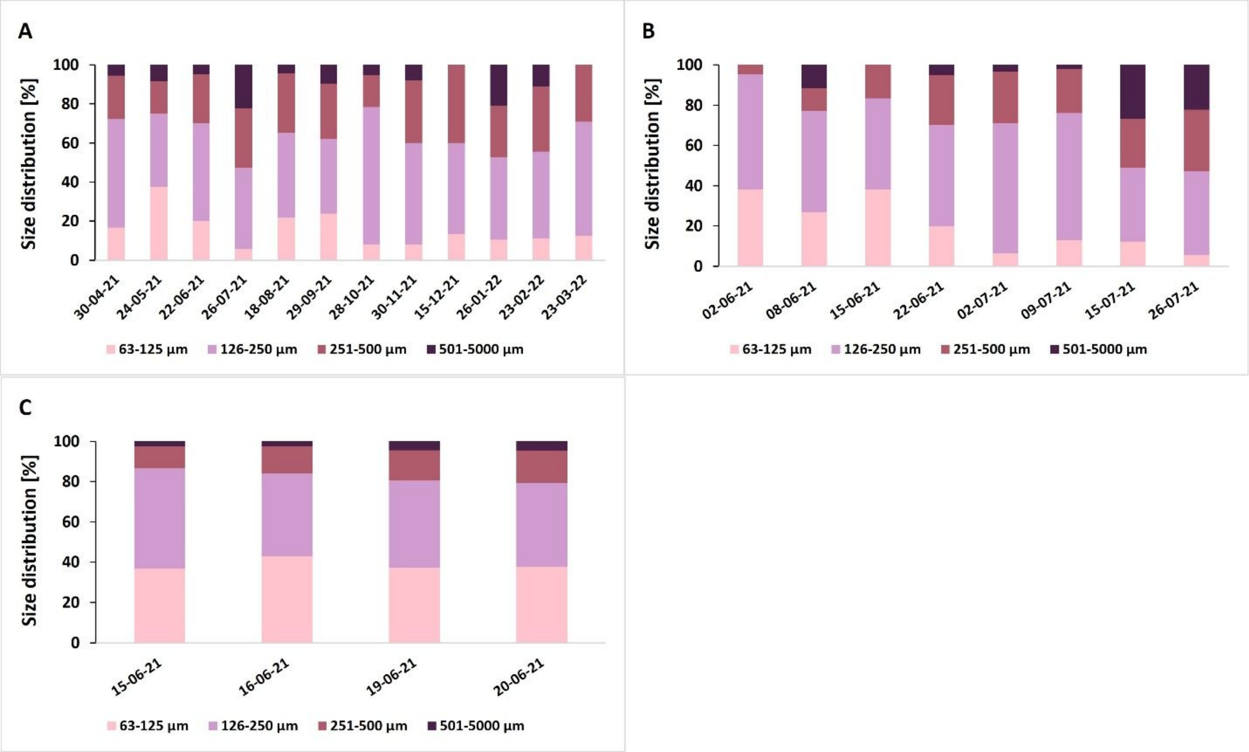

Figure S6: The size ranges of the MP fragments extracted from the samples for: A) monthly sampling, B) weekly sampling and C) hourly sampling as a summary. The size fraction bins reflect standard sediment grain sizes between very fine sand and fine gravel.

S9: Microplastic polymer composition

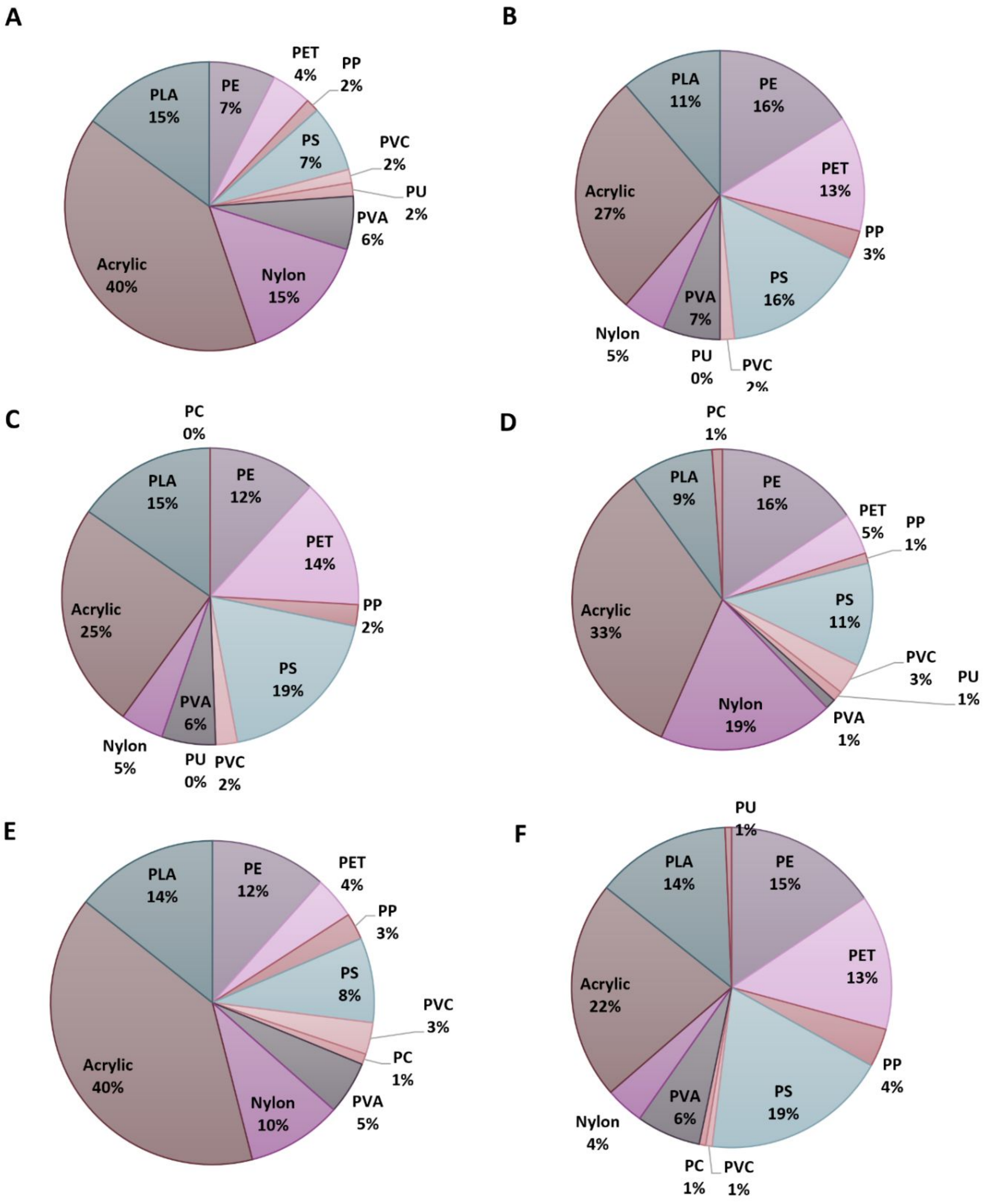

Figure S7: The detailed breakdown of polymer types extracted from the monthly samples: A) Fragments (n = 75), B) fibres (n = 64); the weekly samples: C) Fragments (n = 80) and D) fibres

(n = 90); and from the hourly samples pooled together E) fragments (n = 186) and B) fibres (n = 154).

S10: Stream discharge and microplastic concentration

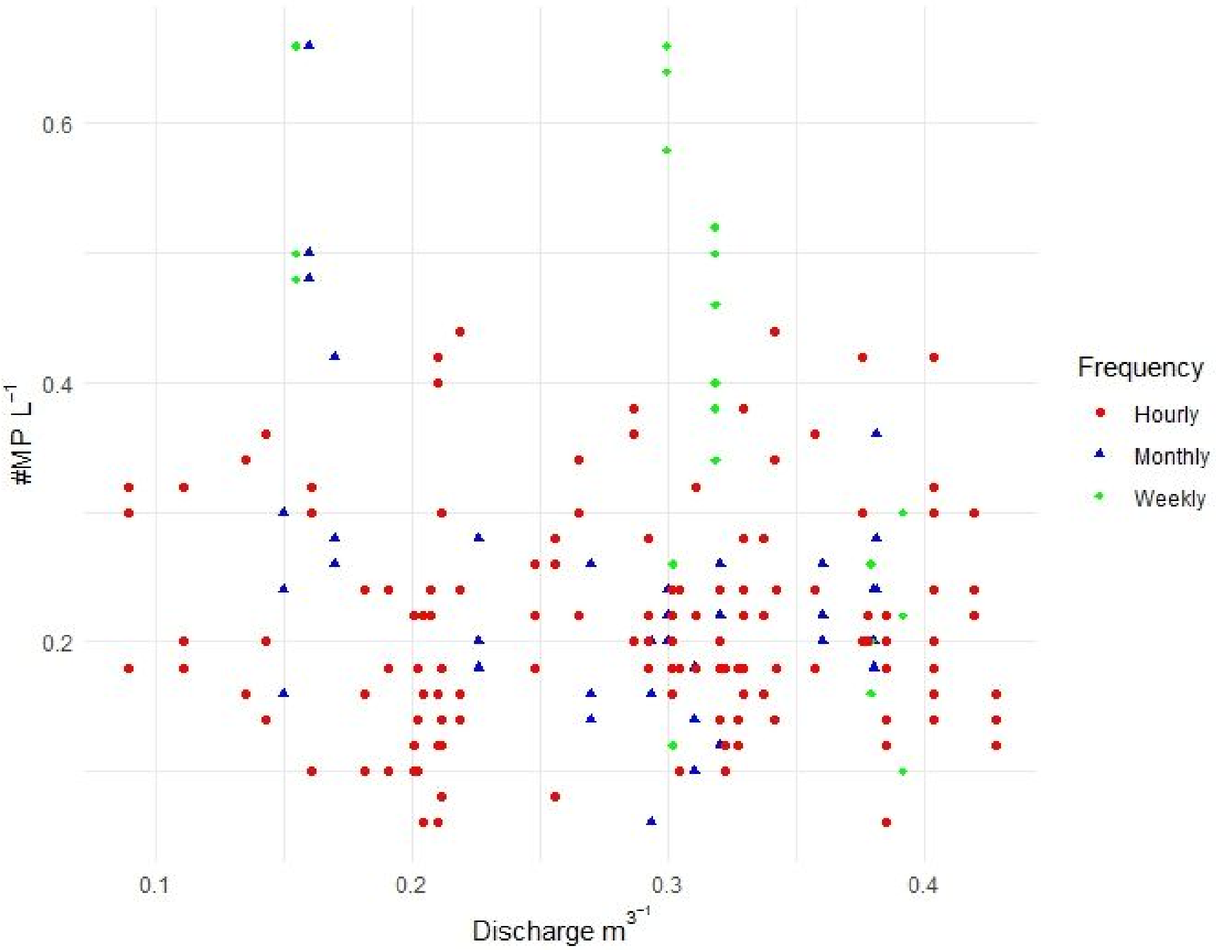

Figure S9: Scatter plot showing microplastic per litre on Y axis (#MP L^{-1}) and measured stream discharge on X-axis (m^3 s^{-1}). Different sampling frequencies are shown with different shaped markers: triangle: 12-monthly, circular: sub-daily and diamond weekly.

S11. Assessing the coefficient of variation for hourly microplastic concentrations

To further assess and understand the variability of the collected hourly MP samples, and to assess how many hourly samples may be needed to characterize the in-stream MP concentration and obtain values close to the daily average (i.e., the mean of the 12 hourly samples per day), we determined all unique combinations of 3, 5, 8, and 10 samples for each of the four 12-hour sampling days. For each of these unique combinations we computed the mean value and then compared these values to the actual daily average (12 samples). To better represent the variation of these combinations of different mean values, we calculated the coefficient of variation (CV) for each of the combinations as follows: (Standard Deviation of CVs divided by the mean) *100%. Higher CV values suggest higher variability and dispersion of the population around the population mean. This allowed us to visualise the dispersion around the means for the unique combinations and their respective range.

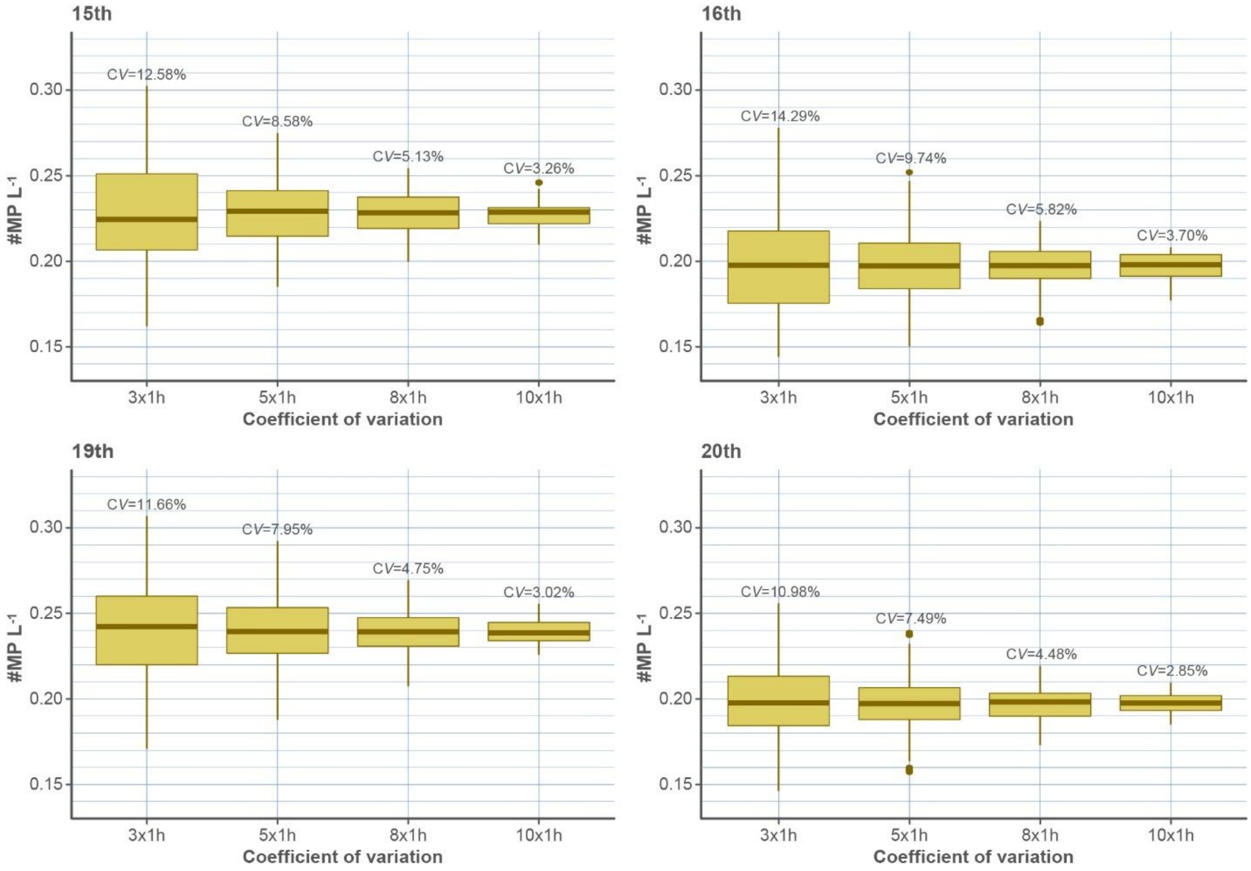

Figure S10: The coefficient of variation of MP concentration means for all randomly chosen 3x1h, 5x1h and 10x1h combinations for days when hourly samples were collected. Larger CV values indicate a higher dispersion level around the mean.

1. Margenat, H., Nel, H.A., Stonedahl, S.H., Krause, S., Sabater, F., Drummond, J.D., *Hydrologic controls on the accumulation of different sized microplastics in the streambed sediments downstream of a wastewater treatment plant (Catalonia, Spain).* Environmental Research Letters, 2021. **16**.
2. Kukkola, A., et al., *Easy and accessible way to calibrate a fluorescence microscope and to create a microplastic identification key.* MethodsX, 2023. **10**: p. 102053.
3. Kelleher, L., et al., *Microplastic accumulation in endorheic river basins – The example of the Okavango Panhandle (Botswana).* Science of The Total Environment, 2023. **874**: p. 162452.
4. Munno, K., et al., *Increasing the Accessibility for Characterizing Microplastics: Introducing New Application-Based and Spectral Libraries of Plastic Particles (SLoPP and SLoPP-E).* Analytical Chemistry, 2020. **92**(3): p. 2443-2451.
